# The Discoverer of Chloroform

**Published:** 1849-02

**Authors:** 


THE DISCOVERER OF CHLOROFORM.

It has been generally stated that Chloroform was discovered, about the same time, by Soubeiran of France, and Liebig of Germany, and the claims of an American to the discovery have been strangely overlooked by nearly every writer on the subject. We take great pleasure in mentioning the name of *Dr. Samuel Guthrie*, late of Sackets Harbor, in connection with the discovery. Not only did Dr. Guthrie procure chloroform about the same time that it was obtained in Europe, but he was the first to publish an account of its therapeutical effects as a diffusible stimulus. This credit was lately awarded to him by a committee of the Medico-Chirurgical Society of Edinburgh. Dr. Guthrie died on the 19th of October last, aged sixty-six years, but not until he had seen his discovery invested with a dignity and an importance, of which he could have had but little conception when he announced it to the profession, in 1832. He was the inventor of the percussion powder, and the only manufacturer of that article in the United States. He was endowed with intellectual powers of a high order, and was a devoted student of nature, and eminent in his profession, until drawn away from it by pursuits in which he felt a greater pleasure. Chemistry was his favorite study, and in connection with chloroform his name is likely to be familiar in both hemispheres.

